# Survival Pathways Are Differently Affected by Microgravity in Normal and Cancerous Breast Cells

**DOI:** 10.3390/ijms22020862

**Published:** 2021-01-16

**Authors:** Noemi Monti, Maria Grazia Masiello, Sara Proietti, Angela Catizone, Giulia Ricci, Abdel Halim Harrath, Saleh H. Alwasel, Alessandra Cucina, Mariano Bizzarri

**Affiliations:** 1 Department of Experimental Medicine, Sapienza University of Rome, 00161 Rome, Italy; noemi.monti@uniroma1.it; 2 Systems Biology Group Lab, Sapienza University of Rome, 00161 Rome, Italy; 3 Department of Surgery “Pietro Valdoni”, Sapienza University of Rome, 00161 Rome, Italy; mariagrazia.masiello86@gmail.com (M.G.M.); sara.proietti@uniroma1.it (S.P.); alessandra.cucina@uniroma1.it (A.C.); 4 Department of Anatomy, Histology, Forensic-Medicine and Orthopedics, Section of Histology and Embryology, Sapienza University of Rome, 00161 Rome, Italy; angela.catizone@uniroma1.it; 5 Department of Experimental Medicine, Università degli Studi della Campania “Luigi Vanvitelli”, 80138 Naples, Italy; giulia.ricci@unicampania.it; 6 Department of Zoology, College of Science, King Saud University, Riyadh 11451, Saudi Arabia; halim.harrath@gmail.com (A.H.H.); salwasel@ksu.edu.sa (S.H.A.); 7 Azienda Policlinico Umberto I, 00161 Rome, Italy

**Keywords:** microgravity, apoptosis, cytoskeleton, Akt, ERK, vinculin, survival pathways, space biomedicine

## Abstract

Metazoan living cells exposed to microgravity undergo dramatic changes in morphological and biological properties, which ultimately lead to apoptosis and phenotype reprogramming. However, apoptosis can occur at very different rates depending on the experimental model, and in some cases, cells seem to be paradoxically protected from programmed cell death during weightlessness. These controversial results can be explained by considering the notion that the behavior of adherent cells dramatically diverges in respect to that of detached cells, organized into organoids-like, floating structures. We investigated both normal (MCF10A) and cancerous (MCF-7) breast cells and found that appreciable apoptosis occurs only after 72 h in MCF-7 cells growing in organoid-like structures, in which major modifications of cytoskeleton components were observed. Indeed, preserving cell attachment to the substrate allows cells to upregulate distinct Akt- and ERK-dependent pathways in MCF-7 and MCF-10A cells, respectively. These findings show that survival strategies may differ between cell types but cannot provide sufficient protection against weightlessness-induced apoptosis alone if adhesion to the substrate is perturbed.

## 1. Introduction

Microgravity is one of the major challenges facing astronauts during spaceflight. Exposure to microgravity has been associated with various significant changes in the organization and function of several tissues [1]. Namely, numerous reports have highlighted that weightlessness—experienced in real (i.e., on board the International Space Station) or in simulated microgravity (i.e., performed with specific tools, like the random positioning machine, RPM)—may foster programmed cell death in living cells [2,3,4]. Cell death may be promoted by activating different pathways, including apoptosis, autophagy, and anoikis, and controversial results and discrepancies have been described [5]. Divergences among findings gathered to date may depend on differences in experimental design, technical tools (especially when simulated weightlessness is considered), and cell types [6]. Furthermore, cells growing in microgravity are frequently partitioned into two different clusters—cells adhering to the substrate or, alternatively, organized into floating, organoid-like structures [7]—and it is likely that these two different phenotypes may respond in a very different way to weightlessness. Indeed, microgravity-dependent loss of adherence and cytoskeleton remodeling in floating cells can increase apoptosis when compared to cells that are growing adherent to the substrate [8]. Given that the rate of apoptosis is usually estimated by mixing cells displaying different adhesion capabilities when exposed to weightlessness, it is conceivable that the measured incidence of apoptosis is in fact an “average” obtained from different cell samples, each one displaying a specific cell death program. Additionally, as cells seem to efficiently cope with the stressful condition of microgravity by self-limiting the overall incidence of apoptosis when exposed for long periods [9], it can be hypothesized that living organism can activate pro-survival strategies. Undoubtedly, these are very relevant questions for properly planning scientific inquiries related to space biomedicine studies. Moreover, different responses triggered by normal or cancerous cells in the presence of biophysical stressful conditions could, in principle, suggest useful insights for a better appraisal of apoptotic-related processes occurring in different pathological conditions, including cancer.

To address such issues, we planned to investigate apoptosis and survival pathways in both normal (MCF10A) and neoplastic (MCF-7) breast cells cultured in RPM versus cells growing in normal gravity condition (1 *g*). These populations display different adhesive responses to the substrate, as MCF-7 cells are partitioned into adherent and nonadherent clusters when cultured in simulated microgravity [6]. Our findings show that the early apoptotic response is actively counteracted by the concomitant activation of two different survival pathways—ERK and Akt—occurring in MCF10A and MCF-7 cells, respectively. However, at later times, a significant increase in apoptosis can be recorded in non-adherent MCF-7 cells only, indicating that loss of adherence and the concomitant disruption of cytoskeleton promoted by microgravity can ultimately overcome the pro-survival strategy enacted by cancerous MCF-7 cells.

## 2. Results

### 2.1. Microgravity Induces the Emergence of Two Stable Populations in Both MCF10A and MCF7

**Figure 1:**
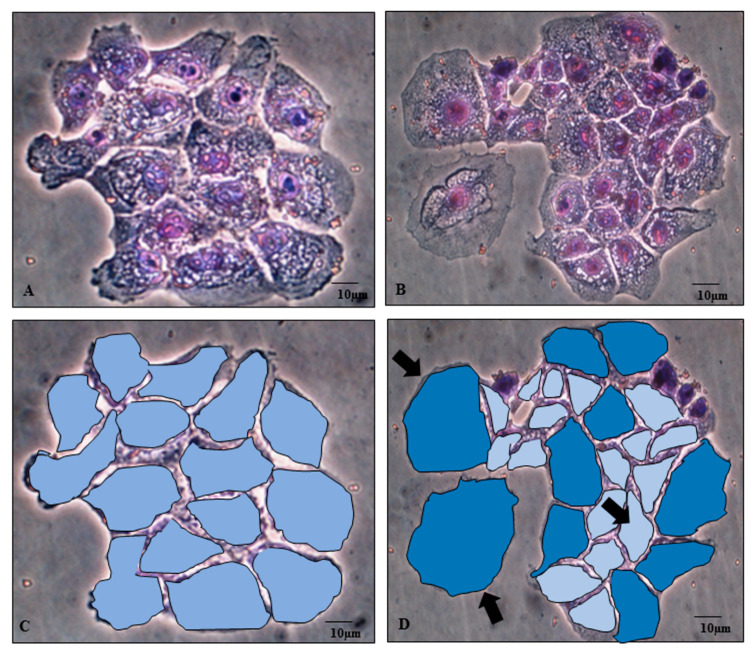
Microphotographs of MCF-10A by optical microscopy. MCF-10A cell line in the on-ground control condition (**A**,**C**) and exposed to microgravity (**B**,**D**) for 24 h. Magnification ×320. The images have been cut out (**C**,**D**) to highlight differences in both area and cell profile. Cropped images show that in microgravity, two distinct populations emerge (evidenced in blue and light blue), displaying different fractal dimension and roundness values, as reported in Table 1. Scale bar 10 µm.

**Figure 2:**
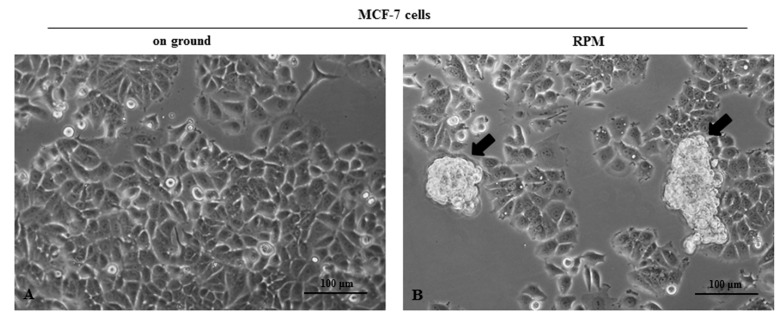
Microphotographs of MCF-7 by optical microscopy. MCF-7 cell line in the on-ground control condition (**A**) and exposed to microgravity (**B**) for 24 h. MCF7 cells in microgravity were distributed into two populations. The first, represented by floating-clump (random positioning machine (RPM)-CLUMP) cells, are indicated by black arrows, and the second are composed adherent cells (RPM-AD.) Magnification ×100. Scale bar 100 µm.

Microgravity induces astonishing modifications in cell morphology. Shape changes undergo early after microgravity exposition and are clearly recognizable after six hours (data not shown). After 24 h of microgravity conditioning, two different morphologic phenotypes spontaneously emerge, and cells are partitioned almost equally (49% versus 51%) into two phenotypically different populations. MCF10A cells split into two distinct morphological phenotypes, recognizable for size and shape features (large and small cells, RPM-L and RPM-S, respectively), growing adherently to the substrate (Figure 1). In MCF-7 cells, one population is represented by flat, spindle cells adhering to the substrate (RPM-AD), while clumps or rounded cells (RPM-CLUMP) constitute the second subset, floating in the culture medium and associated with discrete cluster-resembling organoid-like structures (Figure 2A,B). A cell’s phenotypes emerging after exposure to weightlessness have been demonstrated to be stable and viable over long periods (>72 h, data not shown), and the relative proportion of the two morphological clusters remains invariant for the full period of observation, suggesting that the intrinsic dynamics between the two populations are operating close to the equilibrium. This behavior has been previously observed in other types of breast cancer (MDA-MB-231) [10] as well as in cells obtained from different tissues (bones, lymphocytes, and thyroids) [11,12].

### 2.2. Morphological Parameters

To assess how these changes are mirrored by simultaneous modifications in quantitative shape parameters, we investigated roundness (RO) and fractal dimension (FD) in both cell types after 24 h of weightlessness exposition. In both MCF10A and MCF-7, the two cell clusters enacted by exposure to simulated microgravity show significant differences in roundness and fractal dimension (Table 1). Differences can still be observed when these parameters are compared with those measured in cells growing in normal gravity. Namely, by cutting out MCF10A growing in normal gravity or in simulated microgravity, respectively, one can easily appreciate the reported morphological differences (Figure 1). Overall, such findings confirm that weightlessness promotes the partitioning of cells into distinct phenotypes, thus influencing the subsequent fate and behavior of each one of these clusters.

**Figure 3:**
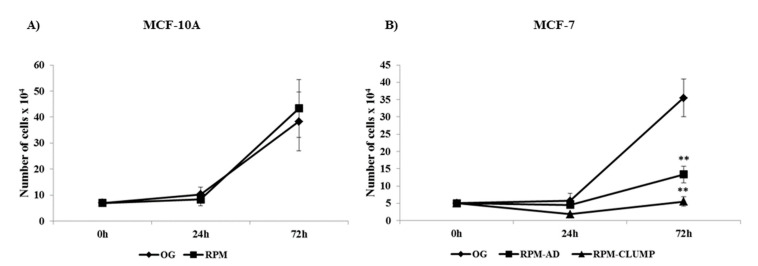
Effect of microgravity on proliferation of MCF-10A (**A**) and MCF-7 (**B**) cells at 24 and 72 h after exposition to weightlessness. Results showing the number of cells ×10^4^ represents the mean value ± SD of three independent experiments. ** *p* < 0.01 vs. on ground (OG) by ANOVA followed by Bonferroni post-test.

Six studies carried out on cells cultured in simulated weightlessness have reported small, albeit significant, changes in growth and cell cycle distribution. Therefore, we assessed those parameters in both cell populations under scrutiny. In MCF10A, no significant differences were found in cell cycle distribution among cells cultured in normal or reduced gravity (data not shown), while in MCF-7 cells, a striking increase in both S and G2M phase was observed for RPM-CLUMP cells, as previously reported [6]. Proliferation, at 24 and 72 h after exposition to weightlessness was almost unchanged in MCF10A (Figure 3A). As expected, a significant decrease in growth rate was observed in MCF-7, especially in the RPM-CLUMP cluster at 72 h (Figure 3B). Overall, these findings suggest that floating cells experience appreciable stress, which is subsequently followed by an increase in the G2M fraction that favors cell growth arrest and consequent apoptosis enhancement, as previously observed in other experimental models of cellular stress [13].

### 2.3. Cytoskeleton Changes

Changes in cytoskeleton (CSK) architecture have been observed during simulated and real microgravity, as early as after a few minutes [14]. Indeed, CSK self-organized processes are highly sensitive to the gravity field [15], and CSK proteins are deregulated even before any change in gene expression pattern can be recorded [16]. CSK changes are instrumental in driving progressive modifications in cell shape, and some of these architectural alterations play a pivotal role in triggering a wide range of death processes, including apoptosis and anoikis [17]. Accordingly, in recent decades, the drug-mediated interference with CSK remodeling has been actively pursued to find a successful strategy of cancer treatment [18]. Therefore, we explored CSK changes in both MCF10A and MCF-7 cells in microgravity. Overall, both cell types underwent appreciable CSK modifications, although the most profound were recorded—as expected—in floating MCF-7, in which cell detachment plays a pivotal role in promoting a number of different programmed cell death pathways, including anoikis.

### 2.4. F-actin

**Figure 4:**
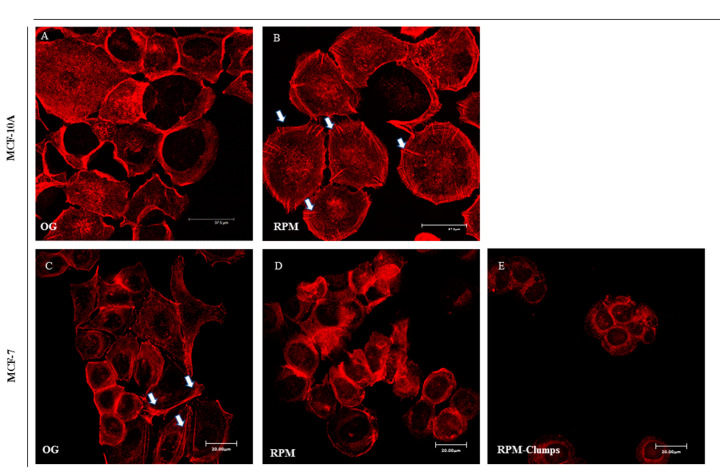
Confocal images of F-actin distribution pattern in MCF-10A (**A**,**B**) and MCF-7 (**C**–**E**) cultured in normal gravity (OG) and microgravity (RPM) conditions for 24 h. MCF-10A cells cultured in RPM conditions (**B**) show actin bundles orthogonal to the periphery of plasma membrane (arrows; scale bar 37.50 μm), while F-actin fibers appear well-organized in MCF-7 cultured in the on-ground condition (**C**, arrows, scale bar 20 μm). In RPM-AD cells, stress fibers are slightly reduced, while F-actin was mostly localized at the cell border (**D**). On the contrary, in RPM-CLUMPS cells the actin filaments are fragmented and significantly reduced (**E**), mostly positioned at the perinuclear level of floating cells €.

In MCF10A cells exposed to microgravity, F-actin shows a different distribution pattern when compared to cells cultured on ground. Cortical F-actin can be recognized as parallel bundles to plasma membrane in 1*g* cultured cells (Figure 4A), whereas in RPM-cultured MCF-10 cells, actin bundles are orthogonal to the periphery of plasma membrane (Figure 4B, arrows). In MCF-7 cells, after 24 h of simulated microgravity, both RPM-AD and RPM-CLUMP cells display a large rearrangement of F-actin when compared to 1*g* control cells (on ground, OG). In control MCF-7 cells, cytosolic F-actin fibers appear well organized in bundles associated with the cell plasma membrane, with recognizable stress fibers and a well-shaped network of stress fibers in the cytosol (Figure 4C). In RPM-AD cells, stress fibers are slightly reduced, while F-actin is mostly localized at the cell border (Figure 4D). On the contrary, in RPM-CLUMPS cells, the actin meshwork loses its organization, and actin filaments appear short, fragmented, and significantly reduced, mostly recognizable at the perinuclear level of floating cells that form the organoid-like structure, whereas stress fibers are dramatically reduced (Figure 4E).

### 2.5. Microtubules

**Figure 5:**
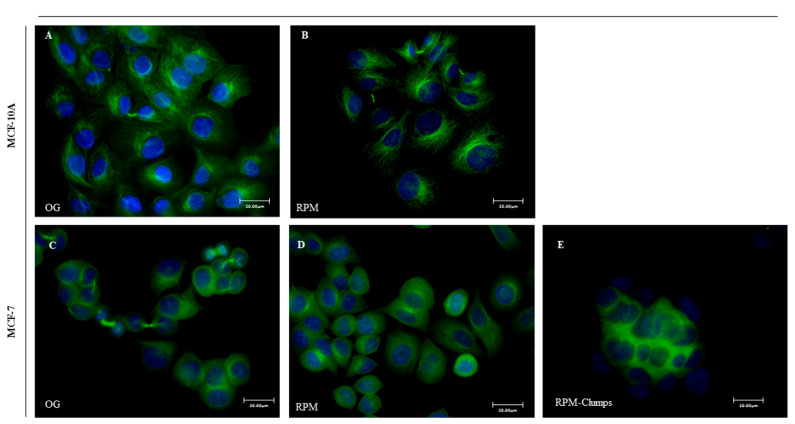
Immunofluorescence microscopy images of alpha-tubulin (in green) distribution pattern in MCF-10A (**A**,**B**) and MCF-7 (**C**–**E**) cultured in normal gravitational (OG) and microgravity (RPM) conditions for 24 h. Nuclei (in blue) were stained with TO-PRO-3 iodide. Scale bar 20 μm.

In both MCF-7 and MC10A cells exposed to weightlessness, we recorded the disappearance of polarized fluorescence associated with the microtubule-organizing center. However, in adherent cells—in MCF-7 as well as in MCF10A—microtubules are still identifiable as filaments that unusually proceed from the perinuclear space toward the cell membrane. Instead, in RPM-CLUMP cells, the tubulin network shows a dramatic disorganization, and tubulin loses its polarization and messily aggregates around the nucleus (Figure 5A–E). Similar findings have already been reported for thyroid cells [19].

### 2.6. Intermediate Filaments: Cytokeratins

**Figure 6:**
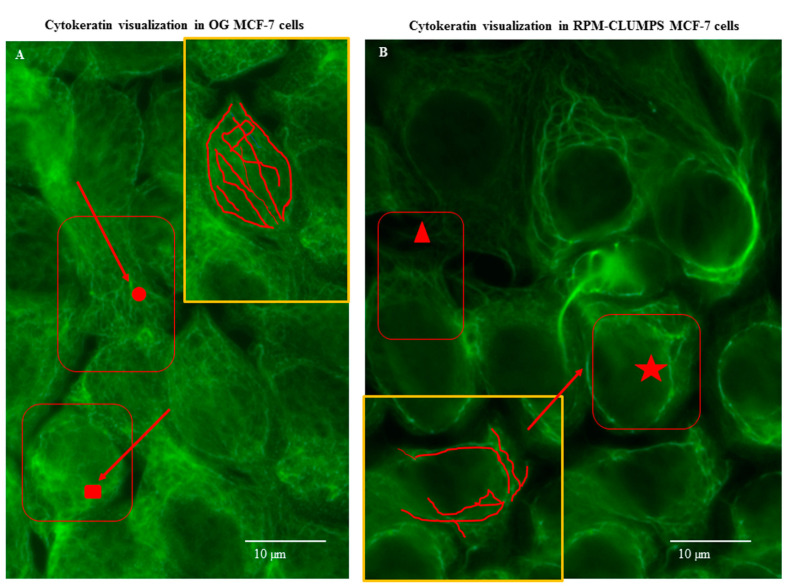
Confocal images of cytokeratin distribution pattern in MCF-7 cultured in normal gravitational (OG) (**A**) and microgravity (RPM-CLUMPS) (**B**) conditions for 24 h. Scale bar 10 µm. ● Desmosomes plaques at the cell–cell junction areas; ▀ perinuclear regions surrounding cell nuclei; 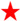 meshes of the cytokeratin network in the perinuclear regions are looser in microgravity than in 1 g control; ▲ plaques reduced both in number and in network density. Within the yellow squares, the distribution of cytokeratins has been magnified. Red arrows indicate the relevant structures under scrutiny.

Cytokeratins (CKs)—composed of intermediate filaments (IF)—are the third most relevant constituent of the cytoskeleton. Usually, the cytokeratin network surrounding the nucleus displays a “net architecture”, resembling a crate in which meshes are regular in size and depicting an “alveolar-like” structure in both MCF10A and MCF-7 cells [20]. Instead, MCF-7 cells exposed to weightlessness exhibit a loosely organized perinuclear cytokeratin network, as previously reported [21]. Yet, while changes were minimally undetectable in MCF10A (data not shown), modifications in the intermediate filament distribution were remarkably evident in MCF-7 cells, especially in the RPM-CLUMP cluster (Figure 6A,B). Given that the intermediate filament organization is instrumental in modulating the effects of external physical stresses [22]—because the cell responds to change in the mechanical environment by fine tuning the aggregation of IFs from concentration fields of the soluble pool—it is tempting to speculate that microgravity-dependent reduction of tensile forces exerted by the *milieu* upon cells could have a significant influence on different biological functions, including apoptosis. Indeed, loosening of the cytokeratin network and degradation of cytokeratin 8/18 have been associated with the early steps of apoptosis in both normal [23] and cancer cells [24]. It is conceivable that disorganization of the cytokeratin meshwork as well as the reduced cytokeratin content—as suggested by the reduced CK density observed in confocal analysis—may contribute to apoptosis in microgravity.

### 2.7. Vinculin

**Figure 7:**
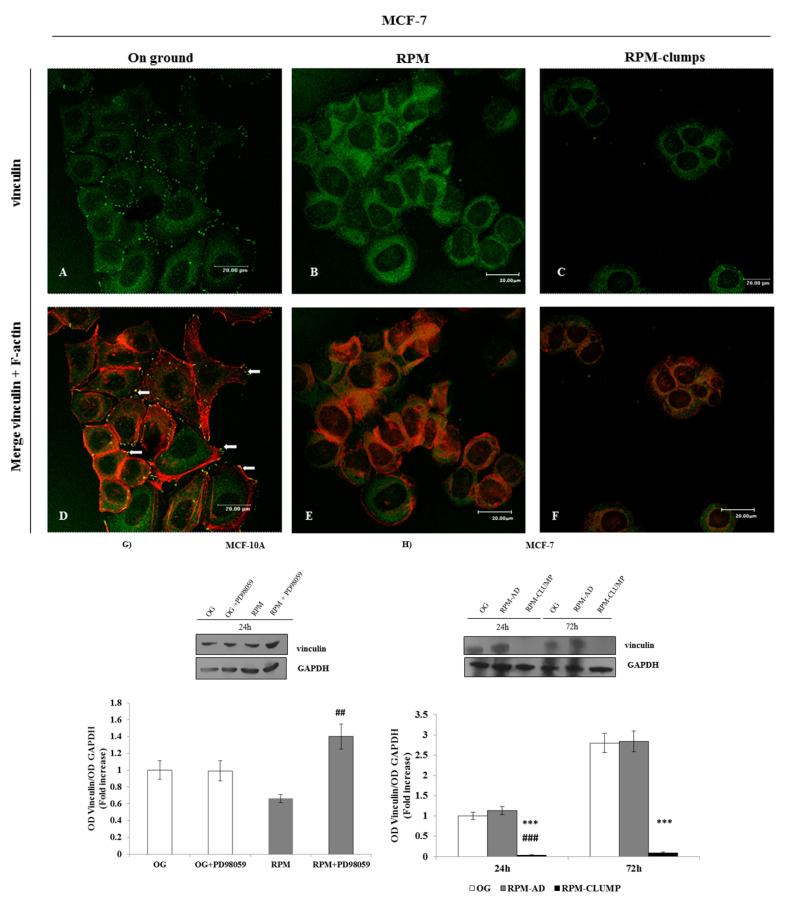
Confocal images of vinculin (green) and F-actin (red) distribution in MCF-7 cultured in normal gravitational conditions (**A**,**D**) and microgravity conditions (**B**,**E**,**C**,**F**) for 24 h. Vinculin distribution is localized nearby focal adhesion (FA) in MCF-7 cultured in the on-ground condition (**A**–**D**, arrows). In MCF-7 RPM adherent cells, vinculin was recognizable within the cytosol and diminished in proximity plasma membrane (**B**,**E**). In MCF-7 RPM-CLUMP cells, vinculin was dramatically reduced and disappeared almost completely from the cytosol and plasma membrane (**C**,**F**). Scale bars 20 µm. Immunoblot bar chart (**G**,**H**) showing the expression of vinculin in MCF-10A (**G**) at 24 h (in correlation with the addition of p-ERK inhibitor PD89059) and in MCF-7 (**H**) in on-ground control cells, RPM adherent cells, and RPM cell clumps at 24 and 72 h. Data show that in MCF10A in microgravity, inhibition of phosphorylated ERK (pERK) leads to an increase in vinculin, while no effects are recorded in 1 g conditions. In MCF-7 cells, vinculin is severely reduced only in RPM-CLUMP cells. Columns and bars represent densitometric quantification of the optical density (OD) of a specific protein signal normalized with the OD values of the GAPDH serving as the loading control. Each column represents the mean value ± SD of three independent experiments. In MCF-10A, ## *p* < 0.01 vs. RPM by ANOVA followed by Bonferroni post-test. In MCF-7, *** *p* < 0.001 vs. OG; ### *p*< 0.001 vs. RPM-AD by ANOVA followed by Bonferroni post-test.

Vinculin is a cytoskeletal protein associated with cell-matrix and cell–cell junctions required for anchoring F-actin to the membrane. In this way, vinculin is indispensable for the mechanical stabilization of cell morphology [25]. Vinculin distribution is severely affected by simulated microgravity, especially in the MCF-7 RPM-CLUMP cluster (Figure 7A–F). In MCF-7 RPM adherent cells, vinculin was chiefly diminished in proximity of focal adhesion (FA), while being still recognizable within the cytosol (Figure 7B). Instead, in floating MCF-7 cells, vinculin was dramatically reduced and disappears almost completely from the cytosol (Figure 7C). Western blot assay showed that vinculin was reduced in both MCF10A and MCF-7 cells (Figure 7G,H) and this reduction is associated with a remarkable decrease in F-actin and stress fibers, together with the disappearance of lamellipodia and pseudopodia formation. These changes are likely instrumental in hampering cells’ adhesion to the substrate. In turn, reduction in cell adhesiveness impairs both FAK activation and actin polymerization, thereby hindering migration. Concurrently, those factors favor cell detachment and the subsequent formation of floating organoid-like structures [26].

It is notable that ERK activity and vinculin levels and the distribution behind the cell membrane are inversely correlated, i.e., a reduction in vinculin appears to be instrumental in enhancing ERK activation [27]. Indeed, ERK was unaffected in adherent MCF-7 cells—in which no significant changes in vinculin were recorded. However, ERK activation was unexpectedly reduced in floating MCF-7 cells, the dramatic decrease in vinculin notwithstanding (Figure 7G,H). Given that ERK activation is tightly coupled with vinculin, we should have expected a “compensatory” increase in ERK activation following the vinculin decrease. The lack of ERK activation in the presence of the decreased vinculin values can be explained by the fact that ERK activation requires the integrity of FAK–integrin complexes, which instead are severely impaired in RPM-CLUMP cells (see above). Consequently, in RPM-CLUMP cells, the almost complete loss of adhesion inhibits the increase in ERK activity that, in principle, should had been triggered by the concomitant disappearance of vinculin.

### 2.8. Integrins

**Figure 8:**
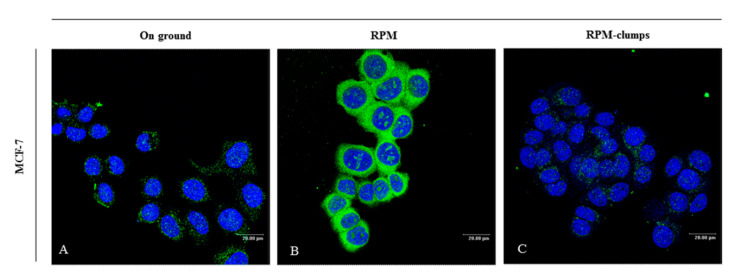
Confocal images of β1-integrin (in green) distribution in MCF-7 cultured in normal gravity (**A**) and microgravity conditions (**B**,**C**) for 24 h. β1-integrin in MCF-7 cells appears localized on the cell membrane both in the on-ground condition and in RPM-cultured cells. β1-integrin shows an increased deposition in RPM-AD (**B**) when compared to control cells cultured on ground. In the RPM-CLUMP cluster, β1-integrin is almost completely absent (**C**). Nuclei (in blue) were stained with TO-PRO-3 iodide. Scale bars 20 µm.

Integrins play a key role in adhesion processes and, conversely, in anoikis, i.e., the programmed cell death occurring because of detachment from the substrate [28]. FAK becomes activated by autophosphorylation upon integrin-mediated cell attachment. Loss of integrin and/or vinculin may hamper their function. The resulting FAK–integrin complex interacts with both ERK and Akt in modulating anti-apoptotic and pro-survival pathways [29]. When compared to cells growing in normal gravity, immunostaining of MCF-7 adherent cells (RPM-AD) shows an increased deposition of β1-integrin on the cell membrane (Figure 8A,B). On the contrary, in the RPM-CLUMP cluster, β1-integrin was almost completely reduced (Figure 8C), as previously observed in thyroid cells growing in simulated weightlessness and forming organoid-like structures [30].

### 2.9. Apoptosis

**Figure 9:**
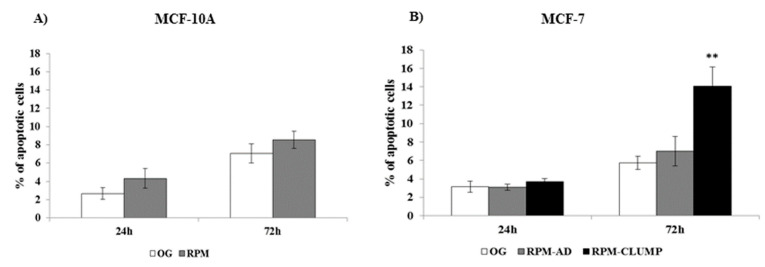
Apoptosis analysis in MCF-10A (**A**) and MCF-7 (**B**) cells at 24 and 72 h after exposition to weightlessness. Bar charts/graphs show the percentage of apoptotic cells (Annexin V+/7-AAD-); each column represents the mean value ± SD of three independent experiments. ** *p* < 0.01 vs. OG by ANOVA followed by Bonferroni post-test. OG—on ground.

A mild, albeit slight increase in apoptosis rate was recorded in MCF10A cells exposed to simulated microgravity at both 24 and 72 h (Figure 9A). Surprisingly, apoptosis was hardly noticed in cancerous MCF-7 cells at 24 h (Figure 9B). However, at 72 h, even MCF-7 cells showed a significant increase in apoptosis, but this effect was limited to RPM-CLUMP cells. This somewhat puzzling behavior indicates that breast cancer cells can efficiently cope with the microgravity stress, at least in the short term. However, at 72 h, apoptosis rate increases steadily, but only in the RPM-CLUMP cluster. This finding is not surprising, as it is well-known that programmed cell death can be successfully induced by disruption of the interactions between normal epithelial cells and the extracellular matrix, despite the fact that this anchorage dependence is reduced in malignantly transformed cells [31]. These observations seem to suggest that some kind of anti-apoptotic processes are enacted in cancerous cells exposed to stressful physical environments, and, at least in the short term, these survival adaptive mechanisms are successful in counteracting cell-death-related processes.

### 2.10. Survival Pathways: Cyclin D1

**Figure 10:**
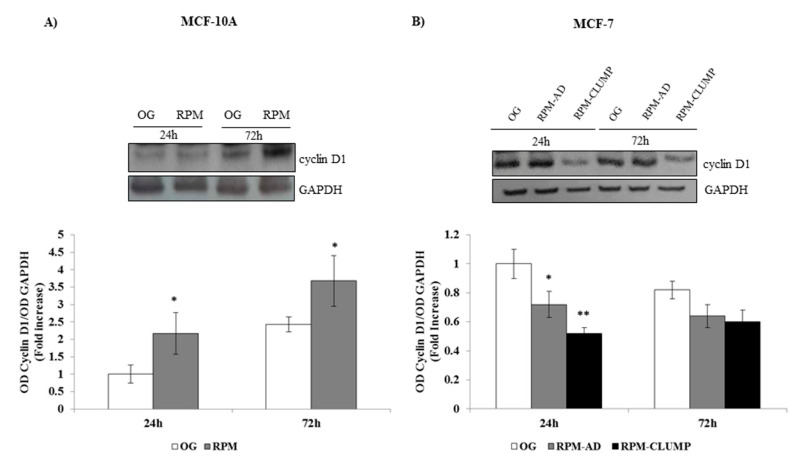
Immunoblot bar chart showing the expression of cyclin D1 in MCF-10A (**A**) and MCF-7 (**B**) in on-ground control cells, RPM adherent cells, and RPM cell clumps at 24 and 72 h. Columns and bars represent densitometric quantification of the optical density (OD) of a specific protein signal normalized with the OD values of the GAPDH serving as the loading control. Each column represents the mean value ± SD of three independent experiments. In MCF-10A, * *p* < 0.05 vs. OG by t test; in MCF-7, * *p* < 0.05, ** *p* < 0.01 vs. OG by ANOVA followed by Bonferroni post-test.

Undoubtedly, cells cultured in simulated microgravity underwent a dramatic remodeling of the cytoskeleton architecture, involving almost all CSK components. These processes promote an outstanding phenotypic transition: cells adopt a new morphological shape and establish entirely new connections between them, as shown by the emergence of organoid-like structures detached from the substrate. In this context, it is not surprising that the apoptosis rate increases because of the relevant changes involving the CSK. Therefore, the right question is not “why do cells in RPM undergo programmed cell death?”, but “why is the apoptosis rate ultimately so limited?”. There are indeed several indices suggesting that cells in microgravity adopt a “survival strategy” by activating some specific biochemical cascades. Indeed, cyclin D1—a molecule amplified in the G1 phase that coordinates the entry, not the S-phase and the subsequent DNA synthesis—is highly expressed in MCF10A exposed to weightlessness and is significantly downregulated in MCF-7 RPM-CLUMP cells (Figure 10A,B). It is notable that cyclin D1 levels correlate with the trend we observed in the proliferation curve (Figure 3A,B), thus supporting the hypothesis of a “survival program” triggered by microgravity. To obtain insights into these processes, we decided to investigate ERK and Akt pathways, which are critically involved in triggering survival and apoptosis resilience.

### 2.11. ERK and Akt

**Figure 11:**
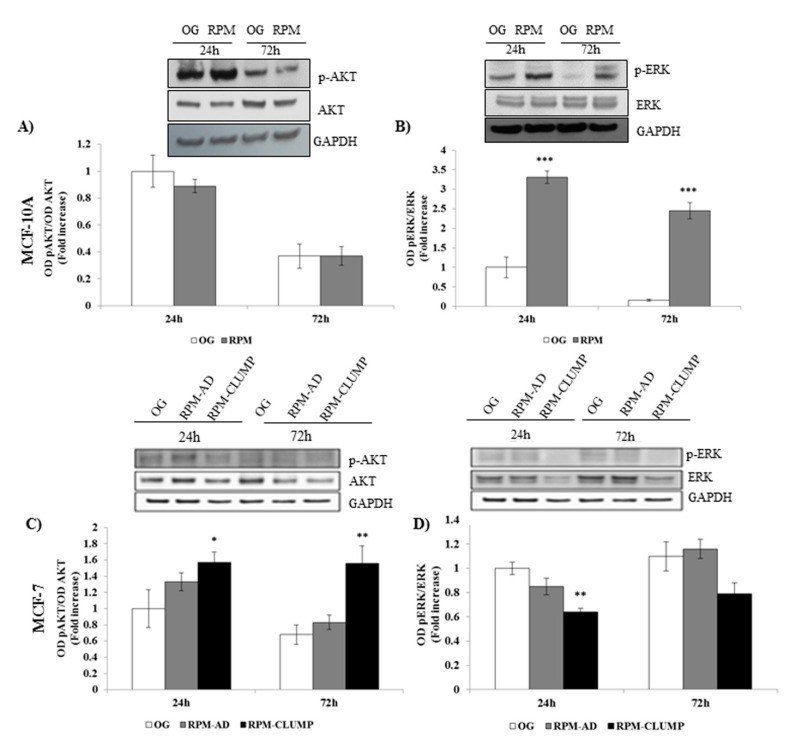
Immunoblot bar chart showing the expression of the phosphorylated AKT (p-AKT)/AKT ratio and p-ERK/ERK ratio in MCF-10A (**A**,**B**) and MCF-7 (**C**,**D**) in on-ground control cells, RPM adherent cells, and RPM cell clumps at 24 and 72 h. Columns and bars represent densitometric quantification of the optical density (OD) of a specific protein signal normalized with the OD values of the GAPDH serving as the loading control. Each column represents the mean value ± SD of three independent experiments. In MCF-10A, *** *p* < 0.001 vs. OG by *t* test; in MCF-7, * *p* < 0.05, ** *p* < 0.01 vs. OG by ANOVA followed by Bonferroni post-test.

We investigated the levels of activated ERK (phosphorylated ERK, pERK) and we found that pERK increases significantly at both 24 and 72 h in MCF10A cells (Figure 11A). On the contrary, pERK was reduced in MCF-7 cells. Conversely, activated Akt (pAkt) does not change in MCF10A, while it increases in MCF-7 cells exposed to microgravity, especially in the RPM-CLUMP cluster of cells (Figure 11B).

**Figure 12:**
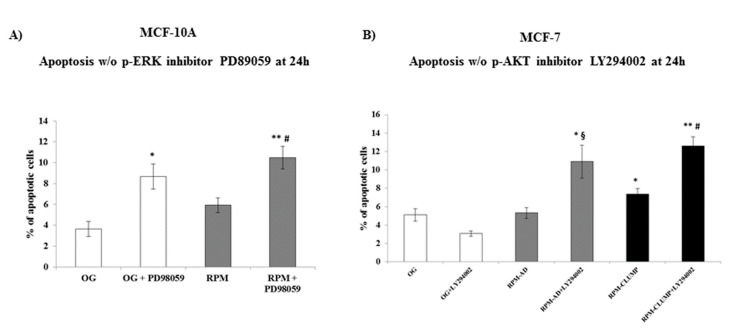
Apoptosis analysis in MCF-10A (**A**) and MCF-7 (**B**) cells at 24 h after exposition to weightlessness w/o the p-ERK inhibitor, PD89059, and w/o the p-AKT inhibitor, LY294002, respectively. Bar charts/graphs show the percentage of apoptotic cells (Annexin V+/7-AAD-); each column represents the mean value ± SD of three independent experiments. In MCF-10A, * *p* < 0.05, ** *p* < 0.01 vs. OG; # *p* < 0.05 vs. RPM by ANOVA followed Bonferroni post-test. In MCF-7 * *p* < 0.05, ** *p* < 0.01 vs. OG; § *p* < 0.05 vs. RPM-AD; # *p* < 0.05 vs. RPM-CLUMP by ANOVA followed by Bonferroni post-test.

Overall, these data seem to suggest that weightlessness triggers a pro-survival response in both cell lines by activating different pathways, i.e., those related to pAkt and pERK in MCF-7 and MCF10A cells, respectively. To assess if the relative increase in both factors could explain how cells in microgravity modulate the apoptotic response, we treated MCF10A and MCF-7 cells with specific inhibitors of pERK and pAkt, respectively. Apoptosis increased up to two- and three-fold in 1*g* and microgravity exposed MCF10A cells (Figure 12A), respectively, upon addition of PD89059, a pERK inhibitor. Conversely, addition of the pAkt inhibitor to MCF-7 exposed to weightlessness increased apoptosis up to three-fold in both RPM-AD and RPM-CLUMP cells (Figure 12B).

**Figure 13:**
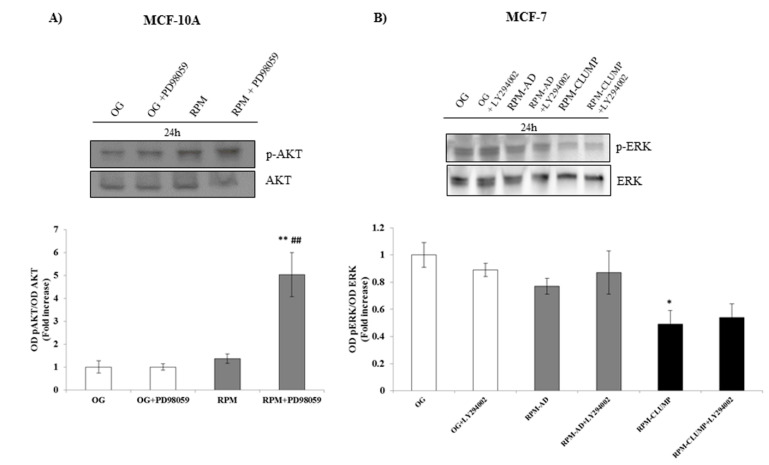
Immunoblot bar chart showing the expression of the p-AKT/AKT ratio and p-ERK/ERK ratio in MCF-10A (**A**) and MCF-7 (**B**) in on-ground control cells, RPM adherent cells, and RPM cell clumps at 24 h w/o the p-ERK inhibitor. PD89059, and w/o the p-AKT inhibitor, LY294002, respectively. Columns and bars represent densitometric quantification of the optical density (OD) of the p-AKT and p-ERK signal normalized with the OD values of AKT and ERK serving as the loading control. Each column represents the mean value ± SD of three independent experiments. * *p* < 0.05, ** *p* < 0.01 vs. OG; ## *p* < 0.01 vs. RPM by ANOVA followed by Bonferroni post-test.

Overall, these findings demonstrated that enhancement of ERK and Akt activity efficiently damped the apoptotic response secondary to microgravity exposure, while the inhibition of both pathways significantly increased apoptosis. The two pathways are not cross-linked in MCF-7 cells, given that the pAkt inhibitor does not modify pERK levels (Figure 13A). Instead, in MCF10A, the inhibition of pERK activity induces a “compensatory” increase in pAkt activity (Figure 13B). These findings show that noncancerous MCF10A cells may paradoxically choose between different survival alternatives by switching from one pathway (pERK) to another one (pAkt), while MCF-7 cells (can) exclusively activate Akt to counteract the microgravity-related pro-apoptotic effects. We have previously observed a similar pattern in breast cancer cell line MDA-MB-231 exposed to weightlessness, in which activated Akt and survivin both increased at 72 h in adherent cells [10].

**Figure 14:**
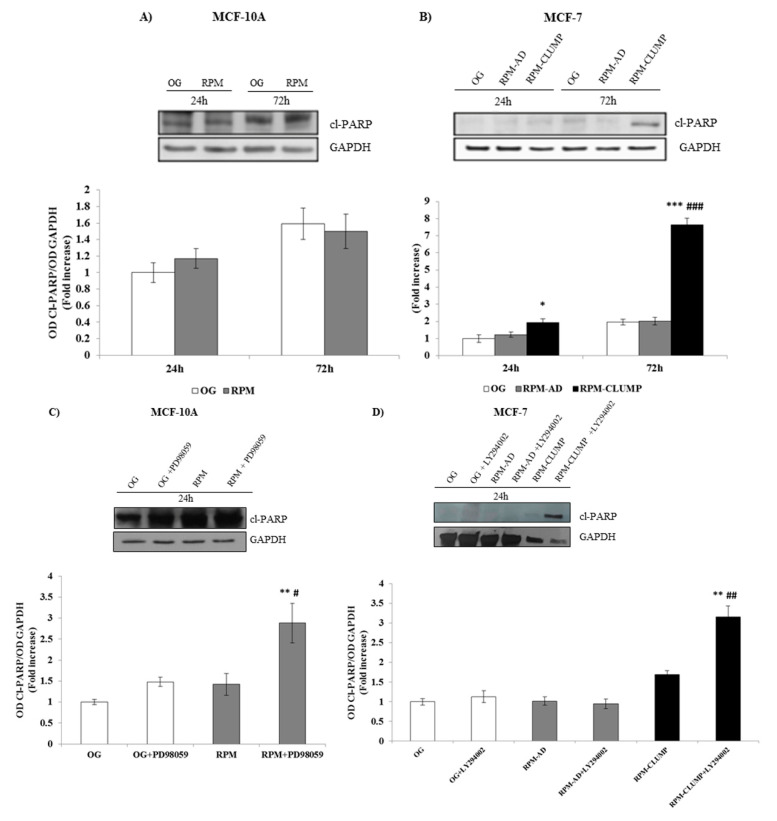
Immunoblot bar chart showing the expression of cleaved poly(ADP-ribose) polymerase (cl-PARP) in MCF-10A (**A**) and MCF-7 (**B**) in on-ground control cells, RPM adherent cells, and RPM cell clumps at 24 and 72 h. Columns and bars represent densitometric quantification of the optical density (OD) of a specific protein signal normalized with the OD values of the GAPDH serving as the loading control. Each column represents the mean value ± SD of three independent experiments. * *p* < 0.05; *** *p* < 0.001 vs. OG; ### *p* < 0.001 vs. RPM-AD by ANOVA followed by Bonferroni post-test. Immunoblot bar chart showing the expression of cl-PARP in MCF-10A (**C**) and MCF-7 (**D**) in on-ground control cells, RPM adherent cells, and RPM cell clumps at 24 h w/o the p-ERK inhibitor, PD89059, and w/o the p-AKT inhibitor, LY294002, respectively. Columns and bars represent densitometric quantification of the optical density (OD) of a specific protein signal normalized with the OD values of the GAPDH serving as the loading control. Each column represents the mean value ± SD of three independent experiments. In MCF-10A, ** *p* < 0.01 vs. OG; # *p* < 0.05 vs. RPM by ANOVA followed by Bonferroni post-test. In MCF-7, ** *p* < 0.01 vs. OG; ## *p* < 0.01 vs. RPM-CLUMP by ANOVA followed by Bonferroni post-test.

These findings were further confirmed when effectors of apoptosis was considered. The enzyme poly(ADP-ribose) polymerase (PARP), whose expression is triggered by DNA strand breaks, represents a main substrate for the activity of caspases, which inactivate PARP through selective cleavage, releasing cleaved PARP (Cl-PARP). In fact, Cl-PARP levels are usually considered a valuable marker for assessing apoptosis [32]. As expected, Cl-PARP levels were only slightly increased in both MCF10A and MCF-7 cells, whereas we observed a four-fold increase in Cl-PARP values after 72 h of weightlessness in RPM-CLUMP MCF-7 cells, mirroring the apoptosis values estimated in those cells (Figure 14A,B). By adding pERK and pAkt inhibitors to MCF10A and MCF-7 cells, respectively, we observed that Cl-PARP increased significantly in both cases, confirming that activation of ERK and Akt pathways markedly decreases PARP cleavage enacted by microgravity exposure (Figure 14C,D).

### 2.12. Proteins That Regulate Apoptosis: Bad, Bax, Bcl-2

**Figure 15:**
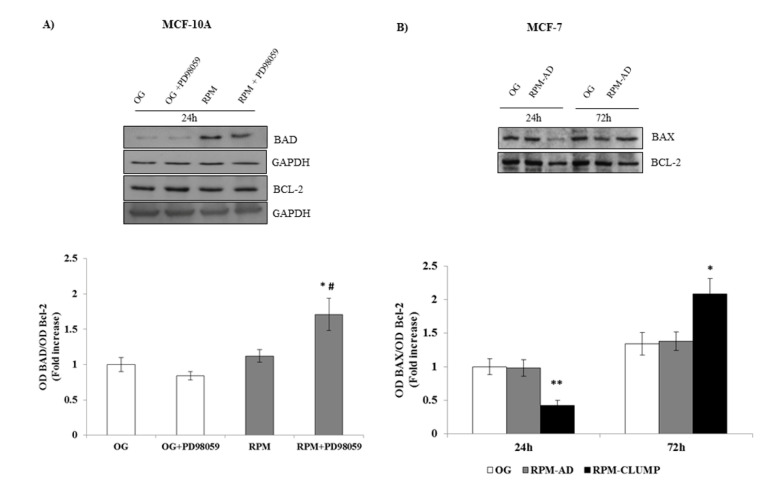
Immunoblot bar chart showing the expression of the Bad/Bcl-2 ratio w/o the p-ERK inhibitor, PD89059, in MCF-10A (**A**) and the Bax/Bcl-2 ratio MCF-7 (**B**) in on-ground control cells, RPM adherent cells, and RPM cell clumps at 24 h. In MCF-10A, columns and bars represent densitometric quantification of the optical density (OD) of a specific protein signal normalized with the OD values of the GAPDH serving as the loading control. In MCF-7, Bax and Bcl-2 were blotted onto the same membrane; thus, Bcl-2 was used as the loading control. Each column represents the mean value ± SD of three independent experiments. In MCF-10A, * *p* < 0.05 vs. OG; # *p* < 0.05 vs. RPM by ANOVA followed by Bonferroni post-test. In MCF-7, * *p* < 0.05, ** *p* < 0.01 vs. OG e vs. RPM-AD by ANOVA followed by Bonferroni post-test.

Members of the Bcl-2 family of regulator proteins that regulate cell death, by either inhibiting (anti-apoptotic) or inducing (pro-apoptotic) apoptosis, include Bax, Bad and Bcl-2 proteins, to mention just a few [33]. Namely, Bax and Bak are believed to initiate apoptosis by forming a pore in the mitochondrial outer membrane that allows cytochrome c to escape into the cytoplasm and activate the pro-apoptotic caspase when the Bad/Bcl-2 and/or Bax/Bcl-2 ratios increase up to 1 [34]. Their involvement allows us to suggest that the intrinsic apoptotic pathway is enacted during microgravity. The Bad/Bcl-2 ratio is not significantly modified in MCF10A cells at 24 h when the apoptotic rate is “unexpectedly” low. However, after the addition of the pERK inhibitor PD98059, Bad increases steadily, while Bcl-2 remains unaffected (Figure 15A), suggesting that ERK activity is instrumental in downregulating Bad rather than in stimulating Bcl-2 activity. In MCF-7 cells, Bax is significantly reduced at 24 h, thus explaining why no relevant apoptosis can be found at this stage. However, at 72 h, when a dramatic increase in apoptosis is observed in RPM-CLUMP MCF-7 cells, a concomitant rise in Bax and in the Bax/Bcl-2 ratio can be observed (Figure 15B). It is worth noting that the anti-apoptotic Bcl-2 protein—when not inhibited by Bax or Bad—antagonizes cytochrome c release through the mitochondrial pore and inhibits activation of the cytoplasmic caspase cascade. This explains why we did not notice an increase in apoptosis at 24 h, when the Bax/Bcl-2 ratio is significantly lower than in control or in RPM-AD cells. Conversely, when the apoptosis rate increases at 72 h, the Bax/Bcl-2 ratio significantly increases.

### 2.13. Survival Cascades Downstream of ERK/Akt Activation

**Figure 16:**
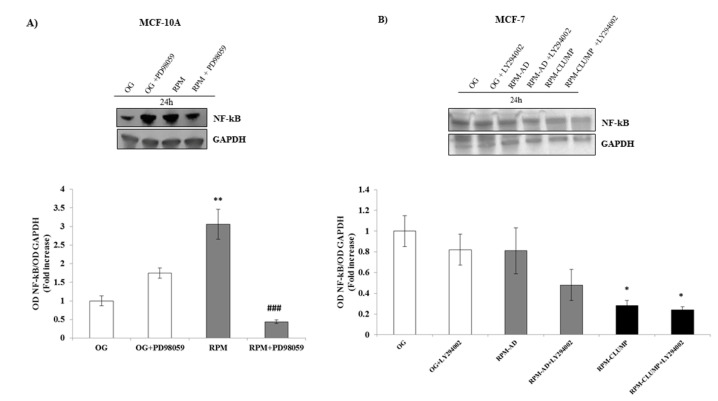
Immunoblot bar chart showing the expression of NF-kB in MCF-10A (**A**) and in MCF-7 (**B**) in on-ground control cells, RPM adherent cells, and RPM cell clumps at 24 h w/o the p-ERK inhibitor, PD89059, and w/o the p-AKT inhibitor, LY294002, respectively. Columns and bars represent densitometric quantification of the optical density (OD) of a specific protein signal normalized with the OD values of the GAPDH serving as the loading control. Each column represents the mean value ± SD of three independent experiments. In MCF-10A, ** *p* < 0.01 vs. OG; ### *p* < 0.001 vs. RPM by ANOVA followed by Bonferroni post-test. In MCF-7, * *p* < 0.05 vs. OG by ANOVA followed by Bonferroni post-test.

Activation of ERK and Akt-related cascades does not only inhibit apoptosis processes but also enhances survival pathways. The increase in NF-kB and survivin activity plays a pivotal role in favoring cell survival under a variety of chemical and physical stresses [35]. Therefore, we investigated NF-kB and survivin release downstream of ERK and Akt activation in MCF10A and MCF-7 cells, respectively. In MCF10A, NF-kB increases up to three-fold in microgravity, while, after the addition of the pERK inhibitor, NF-kB decreases below the control value recorded in MCF10A cells cultured in 1 *g* (Figure 16A). In MCF-7 cells, as no relevant activity of ERK is observed, NF-kB is slightly reduced in both RPM-AD and more significantly in RPM-CLUMP cells (Figure 16B). Nevertheless, a further prominent decrease can be recorded if these cell clusters are treated with the pAkt inhibitor. These findings suggest that while microgravity per se strives in limiting NF-kB activation in MCF-7 cells, the concurrent increase in pAkt activity efficiently antagonizes such effect. Overall, these data show that ERK and Akt support NF-kB activation under stressful conditions, although the overall impacts reach different levels of effectiveness in the two cell lines considered here.

**Figure 17:**
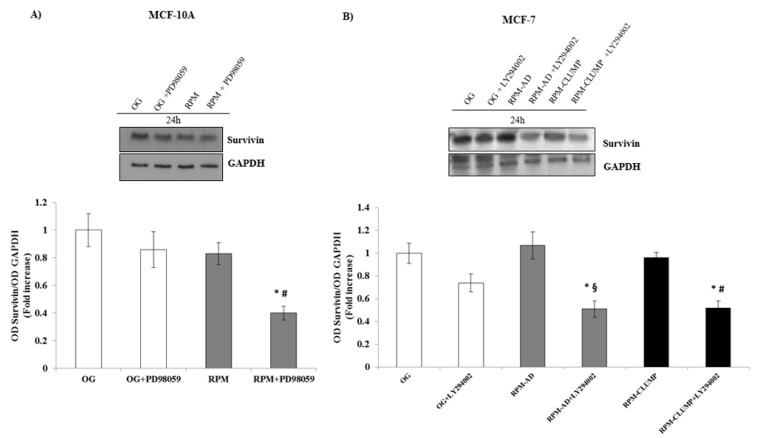
Immunoblot bar chart showing the expression of survivin in MCF-10A (**A**) and in MCF-7 (**B**) in on-ground control cells, RPM adherent cells, and RPM cell clumps at 24 h w/o the p-ERK inhibitor, PD89059, and w/o the p-AKT inhibitor, LY294002, respectively. Columns and bars represent densitometric quantification of the optical density (OD) of a specific protein signal normalized with the OD values of the GAPDH serving as the loading control. Each column represents the mean value ± SD of three independent experiments. In MCF-10A, * *p* < 0.05 vs. OG; # *p* < 0.05 vs. RPM by ANOVA followed by Bonferroni post-test. In MCF-7, * *p* < 0.05 vs. OG; § *p* < 0.05 vs. RPM-AD; # *p* < 0.05 vs. RPM-CLUMP by ANOVA followed by Bonferroni post-test.

Similarly, survivin levels were almost unchanged at 24 h in MCF10A cells exposed to microgravity, while upon addition of the ERK inhibitor, survivin values significantly decreased (−60%, Figure 17A). In MCF-7 cells, at 24 h, survivin increased steadily upon exposure to weightlessness, while dramatically decreasing below the control values after the addition of the pAkt inhibitor (Figure 17B). In agreement with the observed apoptotic rate, survivin decreased at 72 h, mainly in RPM-CLUMP MCF-7 cells, while no significant changes were observed at this time in MCF10A cells (data not shown).

## 3. Discussion

Living cells exposed to microgravity undergo dramatic changes in morphology and biological properties, ultimately culminating into appreciable apoptosis [36,37,38,39], despite some controversial results. Indeed, it has been reported that apoptosis can occur at very different rates, while in some cases, cells appear to be paradoxically protected from programmed cell death during weightlessness [5,40]. These discrepancies can be explained by looking at differences in between cell types, and especially by considering the notion that the behavior of adherent cells dramatically diverges in respect to that of detached cells organized into organoids-like, floating structures. Two main apoptotic pathways have been described: an intrinsic pathway (mostly relying upon events occurring in the mitochondria) and a death-receptor or extrinsic pathway [41], both of them leading to activation of caspases that are responsible for the dismantlement of cells committed to death [42]. However, this canonical model has not been implemented in recent years, as other molecular factors have been recognized to be efficient substitutes for caspases [43], including calpains [44,45], while increasing evidence indicates that remodeling of the cytoskeleton may act as a pivotal trigger in apoptosis initiation upstream of caspases, both in the extrinsic and intrinsic pathway [46]. Moreover, further apoptotic mechanisms—albeit scarcely investigated—have been suspected to play a significant role in triggering cell death in simulated microgravity [27,47].

In our experimental model, we observed that apoptosis affects both MCF10A and MCF-7 cultured in microgravity in different ways. While no significant cell death was noticed at 24 h, a statistically significant increase occurs at 72 h in spite of the fact that such finding is limited to MCF-7 cells detached from their substrate and organized into organoid-like, floating structures. This finding implies that a critical factor in triggering programmed cell death in simulated microgravity exposed cells is represented by cell detachment and the subsequent events that lead to cytoskeleton and shape remodeling. Indeed, we observed that after exposure to microgravity, the architecture of F-actin, microtubules, and intermediate filaments was severely disorganized in organoids-like cell clusters. Minor, albeit significant, changes were also noticed in microgravity-exposed cells growing adherent to the substrate. Looking at those data, we would have expected a higher rate of apoptosis at both early (24 h) and later times (72 h). As this did not happen, we focused on survival pathways to investigate if cell clusters adopt a “survival strategy” to counteract the cytoskeleton disruption induced by weightlessness. It is worth noting that an increase in ERK activity was found to damp apoptosis in cancer cells treated with drugs that disrupt microtubule dynamics and cytoskeleton architecture [48,49]. Moreover, modulation of the cytoskeleton using perturbing drugs promotes mechanically induced signal activation of both ERK and AKT as compensatory mechanisms [50]. Briefly, both Akt and ERK are suspected to be activated to counterbalance the apoptosis induced after cytoskeleton disruption promoted by several chemical/physical factors [51]. Therefore, we investigated such biochemical cascades, and we found that ERK and Akt pathways were selectively and significantly elicited in MCF10A and MCF-7 cells, respectively. Consequently, several molecular factors involved in the apoptotic process were downregulated at early times after weightlessness exposure. Indeed, it has previously been shown that the activation of the ERK pathway has a dominant protecting effect over apoptotic signaling from the death receptors and acts by suppressing activation of caspase machinery [52]. The slowdown of programmed cell death is associated with the concomitant increase in key factors triggering survival pathways, i.e., survivin and NF-kB. On the contrary, by inhibiting ERK in MCF10A cells and Akt activation in MCF-7 cells, we observed that apoptosis was unleashed and increased significantly in these cell types at both early and later times after microgravity exposure (Figure 13A,B). Accordingly, inhibition of ERK/Akt promoted a significant increase in the release of pro-apoptotic markers: Cl-PARP, Bad and Bax. Consequently, survival effectors (NF-kB and survivin) were downregulated after the addition of pAkt and pERK inhibitors.

However, the increase in Akt and ERK is not enough, per se, to successfully antagonize the apoptotic processes driven by weightlessness at later times, given that the loss of adherence may overcome the protective effect provided by the activation of the aforementioned pathways. The integrity of both FAK and integrins is needed to allow the Akt-dependent pathway to display an efficient anti-apoptotic effect, as highlighted by the apoptotic trend that we observed in MCF-7 cells. Indeed, in RPM-AD cells, FAK and integrin can counteract the apoptotic process through an appreciable increase in Akt activity, as apoptosis is still kept at bay at 72 h; on the contrary, in RPM-CLUMP cells—in which integrins are dramatically reduced—apoptosis is markedly increased despite the concomitant extraordinary increase in Akt. However, Akt plays a role in damping the apoptotic increase, given that the treatment with the Akt inhibitor triggers a further relevant apoptotic boost in both RPM-AD and RPM-CLUMP cells. This finding suggests that the current paradigm should be reversed: integrity of the cytoskeleton and of the FAK–vinculin–integrin complexes is probably not necessary to activate the Akt pathway, which ultimately can be triggered even in the absence of such stimuli, but they are mandatory to enable Akt in providing a “protective” effect. In fact, the observed increase in Akt activation is insufficient for blocking the apoptotic process, at least in RPM-CLUMP cells, in which the integrin/cytoskeleton organization is severely compromised. It is recognized that loss of adhesion, namely when experimentally obtained by blocking integrin binding or depriving the cell membrane of their integrin content [53], can suffice per se in inducing cell apoptosis. In our study, the transmission of environmental signals through the FAK–integrin complexes seems mandatory in allowing Akt to properly exert its anti-apoptotic function. In fact, in absence of the substratum anchorage, even higher levels of Akt are insufficient in bestowing cells with an adequate survival shield.

On the contrary, in MCF10A cells, in which substrate adherence is preserved, no significant increase in apoptosis was observed, although the cytoskeleton exhibited remarkable disorganization after exposure to weightlessness. These data suggest that in MCF10A cells, changes in cytoskeleton architecture are in some way counterbalanced by the persistence of integrin-mediated anchorage to the substratum, while the concomitant drop in vinculin significantly enhances ERK activity. Moreover, it is of note that between vinculin and ERK, a further feedback loop may exist, given that—upon microgravity exposure—ERK is enhanced by the reduction in vinculin expression and, in turn, ERK could reinforce vinculin downregulation, as the addition of the pERK inhibitor allows vinculin to be significantly re-expressed.

Overall, such findings outline how relevant the adhesion process is in modulating the survival strategy adopted by cells to antagonize cell death in microgravity [54,55]. The extent to which cytoskeleton and other molecular factors are involved in bestowing a proper anti-apoptotic shield likely depends on cell types and on microenvironmental cues [56,57]. In our model, a critical role is undoubtedly provided by the interplay between integrins, FAK, cytoskeleton filaments, and vinculin. Vinculin reduction is required for activating the ERK-dependent pathway, but the functionality of integrin-FAK complexes is mandatory for enabling both Akt and ERK in counteracting cell death-dependent processes. The fact that different breast cells enact differentiated survival pathways when exposed to a biophysical stress like microgravity deserves further investigations and provides useful suggestions. Furthermore, it is intriguing that cancerous breast cells strive to antagonize cell death by preferentially activating the Akt pathway, whereas normal breast cells activate ERK-dependent biochemical cascades. In both cases NF-kB and survivin, values were enhanced; however, we cannot ignore the fact that other unexplored mechanism could have been selectively promoted. This implies that survival strategies do not overlap in between the two cell types. This statement has huge implications for anticancer treatment, as it suggests that we should focus on the Akt pathway (and upon PI3K, its upstream-driving controller [58]), rather than on ERK-related cascades when pro-apoptotic strategies for controlling breast cancer are envisaged. Finally, the disruption of the cytoskeleton promoted by microgravity can overcome the pro-survival strategy enacted by cancerous nonadherent MCF-7 cells. This observation reinforces the belief that attempts of harnessing the cytoskeleton and the connectivity in between cells and their microenvironment could represent a useful strategy of anticancer treatments.

## 4. Material and Methods

### 4.1. Random Positioning Machine (RPM)

Microgravity conditions were simulated using a Desktop RPM, a particular kind of 3D clinostat manufactured by Dutch Space (B.V. P.O. Box 32070 2303 DB, Leiden, The Netherlands). The angular speed and the inclination of the disk influence the degree of microgravity simulation. RPMs do not actually eliminate the gravity but allow a stimulus rather than a unidirectional or omnidirectional 1 *g* force to be applied. The effects generated by the RPM are comparable to those of real microgravity when the direction changes are faster than the response time of the system to gravity field. The RPM was located in a standard incubator at 37 °C and 5% CO_2_ and connected to the control console through standard electric cables.

### 4.2. Cell Culture

The nontumorigenic epithelial cell line MCF-10A (ATCCCRL-10317) was obtained from LGC Standards S.r.l, MI, Italy; the human hormone-sensitive breast adenocarcinoma cell line MCF-7 (ECACC Cat# 86012803) was obtained from Sigma-Aldrich (St. Louis, MO, USA). Both MCF-10A and MCF-7 were seeded in 25 cm^2^ flasks. MCF-10A cells were grown in Dulbecco’s modified Eagle’s medium/nutrient mixture F12 Ham (Sigma-Aldrich, Merck, Darmstadt, Germany) supplemented with 10% horse serum (Euroclone Ltd., Cramlington, UK) and EGF 500 μg/5mL (Santa Cruz Biotechnologies, Dallas, TX, USA), Hydrocortisone (50 μM), cholera toxin (0.5 mg/mL), insulin (10 mg/mL) (all from Sigma Chemical Co, 3050 Spruce St., St. Louis, MO, USA), and antibiotics (penicillin 100 IU/mL, streptomycin 100 μg/mL, gentamycin 200 μg/mL), all from Euroclone Ltd., Cramlington, UK. MCF-7 were grown in Dulbecco’s modified Eagle’s medium (DMEM) supplemented with 10% fetal bovine serum (FBS) and antibiotics (penicillin 100 IU/mL, streptomycin 100 µg/mL, gentamycin 200 µg/mL; all from Euroclone Ltd., Cramlington, UK).

For microgravity experiments, cells were seeded into Nunc OptiCell Cell Culture Systems, gas-permeable cell culture disks (Thermo Scientific, Rochester, 75 Panorama Creek Dr, Rochester, NY, USA). OptiCells containing subconfluent monolayers were fixed onto the RPM as close as possible to the center of the platform, which was rotated at a speed of 60°/s using the random mode of the machine. On-ground control (1 g static cultures) and RPM cultures were kept in the same humidified incubator at 37 °C in an atmosphere of 5% CO_2_ in air. Experiments were performed for 24 and 72 h. After 24 and 72 h of microgravity exposure, cell clumps swimming in culture supernatants were found, in addition to adherent cells, and separately collected. The three cell populations (on-ground control cells, RPM adherent cells, and RPM cell clumps) were characterized separately.

Similar experiments were performed in the presence of 10 μM PD98059, an inhibitor of ERK phosphorylation (Cell Signaling Technology, Inc., Boston, MA, USA), or 10 μM LY294002, an inhibitor of AKT phosphorylation (Santa Cruz, Biotechnology, Inc. Santa Cruz, CA, USA).

### 4.3. Cell Proliferation

Cells were treated for 24 and 72 h in RPM or kept on ground, and then they were counted with a particle count and size analyzer (Beckman-Coulter, Inc., Fullerton, CA, USA). Three independent experiments in duplicate were performed.

### 4.4. Optical Microscopy

Cell clumps were collected, washed in PBS, and deposited onto a clearly defined area of a glass slide using a Shandon CytoSpin 4 Cytocentrifuge, Thermo Scientific, while maintaining cellular integrity. Cell clumps and adherent and on-ground control cells were fixed in 4% paraformaldehyde for 10 min at 4 °C and photographed with a Nikon Coolpix 995 digital camera coupled with a Zeiss Axiovert optical microscope. The images were obtained with a 100 × or 320 × magnification, saved as TIFF files, and used for image analysis.

### 4.5. Image Analysis

Image analysis was performed on 10 images for each group of cells. As the analysis was performed blindly, the image groups were classified as follows: A (on-ground cells, 24 h), B (RPM adherent cells, 24 h), C (RPM cell clumps, 24 h). In each image, single randomly chosen cells (50 for each group) were contoured with a fine black marker by different researchers, simply scanned, and cataloged according to the time of study. This method was chosen because pathologists are used to correlate the shape the cells acquire with their malignancy by means of morphological, qualitative, and subjective observations. Thus, we decided to perform a semiautomatic analysis, coupling the expertise of researchers with a computerized parameterization method. All the images were processed by Adobe Photoshop CS4. All the pictures (i.e., all the sheets of the groups for each time point) were resized at 2560 × 1920 pixels according to original scale of image acquisition. For each black contoured cell, the edges were refined. Then cells were filled in black, and the threshold was adjusted in order to exclude other cells and backgrounds from the image. For each time point, a single sheet of all of the cells considered was created. To obtain single cell shape parameters (area *A*, roundness, solidity, and fractal dimension FD), ImageJ v1.47h software was used. Then, the software analyzed single cells with the “shape descriptor”. In addition to area *A*, the following were calculated:

Roundness = 4*Aπ*ma√Solidity = *A*CA, (1)

where *A* is the area of the cell, ma is the major axis, and CA is the convex area, namely, the area of the convex hull of the region. The convex hull of a region is the smallest region that satisfies two conditions: (a) it is convex and (b) it contains the original region.

As for FD, it was obtained by means of the box counting method using the FracLac plugin:

FD = lim*ε*→0[1 − log[*Lε*(*C*)]log*ε*] (2)

where *C* is the considered curve, *L* is the length of the curve *C*, and *ε* is the length of the segment used as the unit to calculate *L*.

### 4.6. Annexin V/7-AAD Staining

Cell clumps were collected and centrifuged, and pellets were trypsinized and washed twice with PBS. Adherent cells and ground control cells were trypsinized and washed twice with PBS. The cells were stained with FITC-labeled annexin V/7-AAD (7-aminoactinomycin-D) according to the manufacturer’s instructions (annexin-V/7-AAD kit; Beckman Coulter, Marseille, France). Briefly, a washed cell pellet (5 × 104 cells/mL) was resuspended in 500 μL binding buffer; 10 μL of annexin-V together with 20 μL 7-AAD was added to 470 μL cell suspension. The cells were incubated for 15 min on ice in the dark. The samples were analyzed by flow cytometry. Apoptosis assay was performed three times.

### 4.7. Confocal Microscopy

Cells were fixed with 4% paraformaldehyde for 10 min at 4 °C and washed twice for 10 min with PBS. The cells were permeabilized for 30 min using PBS, 3% BSA, and 0.1% Triton X-100, followed by anti-vinculin (7F9) sc-73614, anti-integrin-β1 sc-8978 (all from Santa Cruz Biotechnology10410 Finnell Street Dallas, Texas 75220 U.S.A.), anti-cytokeratin 8 (DC10) (from Thermo Fisher Scientific 75 Panorama Creek Dr, Rochester, NY 14625, USA), and anti-α-tubulin T5168 (from Sigma Aldrich 3050 Spruce St. Louis, MO, USA). Staining in PBS and 3% BSA was conducted at 4 °C overnight. The cells were washed with PBS and incubated for 1 h at room temperature with the appropriate secondary antibody FITC conjugated (Invitrogen Molecular Probes Eugene, OR, USA). Negative controls were processed in the same conditions as those used in primary antibody staining. For F-actin visualization, rhodamine phalloidin (Invitrogen Molecular Probes Eugene, 1: 40 dilution) was used. Cells were then washed in PBS and mounted in buffered glycerol (0.1 M, pH 9.5). Finally, analysis was conducted using a Leica confocal microscope TCS SP2 (Leica Microsystems Heidelberg GmbH, Mannheim, Germany) equipped with Ar/ArKr and He/Ne lasers. Laser line were at 543 and 488 nm for TRITC and FITC excitation, respectively. The images were scanned under a 40 × oil objective.

### 4.8. Western Blot

Cell clumps were washed twice with ice-cold PBS and resuspended in RIPA lysis buffer (Sigma Chemical Co 3050 Spruce St. Louis, MO, USA). Adherent and ground control cells were washed twice with ice-cold PBS and scraped in RIPA lysis buffer (Sigma Chemical Co.). A mix of protease inhibitors (Complete-Mini Protease Inhibitor Cocktail Tablets, Roche, Mannheim, Germany) and phosphatase inhibitors (PhosStop; Roche, Mannheim, Germany) was added just before use. Cellular extracts were then centrifuged at 8000× *g* for 10 min. The protein content of supernatants was determined using the Bradford assay. For Western blot analysis, cellular extracts were separated on SDS polyacrylamide gels with a concentration of acrylamide specific to the studied proteins. Proteins were blotted onto nitrocellulose membranes (BIO-RAD, Bio-Rad Laboratories, Hercules, CA, USA) and probed with the following antibodies: anti-cyclin D1 (AB-90009) from Immunological Sciences; anti-survivin (number 2808), anti-phospho-AKT (ser473) (number 9271S), anti-AKT (number 9272S), anti-phospho-ERK1/2 (number 9106), anti-cleaved PARP (number 9541), anti-NF-kB #8242, and anti-GAPDH (number 2118), all from Cell Signaling Technology; anti-Bad sc-8044, anti-Bax (sc-493), anti-Bcl-2 (sc-492), anti-ERK1 (sc-94), sc-58326, anti-vinculin (7F9): sc-73614, and anti-integrinβ1 sc-8978, all from Santa Cruz Biotechnology. Antigens were detected with an enhanced chemiluminescence kit (Amersham Biosciences, Little Chalfont Buckinghamshire, UK) according to the manufacturer’s instructions. All Western blot images were acquired and analyzed through Imaging Fluor S densitometer and ChemiDoc Imaging System (Biorad-Hercules). In the graphs, the columns and bars represent densitometric quantification of the optical density (OD) of a specific protein signal normalized with the OD values of the loading control, and they are expressed as fold increase in the control value considered as 1.

### 4.9. Statistical Analysis

Data were expressed as mean ± standard deviation (SD). Data were statistically analyzed with the analysis of variance (ANOVA) followed by the Bonferroni post-test or unpaired two-tailed *t* test. Differences were considered significant at the level of *p* < 0.05. Statistical analysis was performed by using GraphPad Instat software (GraphPad Software, Inc.; San Diego, CA, USA).

## 5. Conclusions

Removal of gravity-related constraints constitutes a relevant systemic stress for living cells. Generally, exposure to simulated microgravity enhances apoptosis, although the cell death rate greatly varies depending on cell types, times of exposition, and culture conditions. Herein. we showed that preserving cell adhesivity to the substrate represents a critical factor in enabling cells to escape from apoptosis. Noticeably, in response to microgravity, both normal and cancerous breast cells activated distinct survival pathways—ERK and Akt in normal and cancer cells, respectively. However, pro-survival countermeasures showed to be only partially effective in cancer cells growing in organoids-like structures detached from the substrate and with dramatically altered cytoskeleton architecture. Not only do these results provide insights regarding the notion that survival pathways enacted by cancer cells differ from those activated by normal cells, but they also contribute to furthering our understanding of how apoptosis in normal tissues [59] can be antagonized and, ultimately, to preserving the health and performance of astronauts exposed to space microgravity.

## Figures and Tables

**Table 1:** Roundness, solidity, and fractal dimension (FD) in MCF-7 and MCF-10A at 24 h and 72 h. Mean values ± SD in on-ground control cells, RPM adherent cells, and RPM cell clumps. ** *p* < 0.01 versus on-ground control cells; *** *p* < 0.001 vs. on-ground control and RPM adherent cells by ANOVA followed by Bonferroni post-test. (Sample size = 50 cells for each group).

**MCF-7 (breast cancer cells, poorly invasive)**									
	**Roundness**	**± SD**		**Solidity**	**± SD**		**Fractal Demension (FD)**	**±SD**	
24 h									
on ground	0.646	±0.0151		0.802	±0.101		1.1560	±0.0356	
RPM (adherent cells)	0.667	±0.139		0.844	±0.130	***	1.1381	±0.0230	
RPM (cell clumps)	0.783	±0.111	***	0.921	±0.034	***	1.1275	±0.1017	***
72 h									
on ground	0.619	±0.149		0.759	±0.100		1.1478	±0.1296	
RPM (adherent cells)	0.654	±0.143	***	0.773	±0.093		1.1633	±0.0333	
RPM (cell clumps)	0.743	±0.130	***	0.897	±0.063	***	1.1385	±0.0248	
**MCF-10A (non tumorigenic mammary)**									
	**Roundness**	**±SD**		**Solidity**	**±SD**		**Fractal Demension (FD)**	**±SD**	
24 h									
on ground	0.709	0.154		0.788	0.259		1.344	0.088	
RPM	0.713	0.135		0.821	0.215		1.371	0.096	**
72 h									
on ground	0.726	0.131		0.856	0.132		1.391	0.092	
RPM	0.711	0.125		0.860	0.090		1.443	0.081	***

## Data Availability

The data that support the findings of this study are available from the corresponding author upon reasonable request.

## Abbreviations

RPM	random positioning machine
RO	calculated roundness
FD	fractal dimension
CSK	cytoskeleton
OG	on ground
CKs	cytokeratins
pERK	phosphorylated ERK
pAkt	phosphorylated AKT
Cl-PARP	cleaved PARP
